# Motor torque measurement of *Halobacterium salinarum* archaellar suggests a general model for ATP-driven rotary motors

**DOI:** 10.1038/s42003-019-0422-6

**Published:** 2019-05-24

**Authors:** Seiji Iwata, Yoshiaki Kinosita, Nariya Uchida, Daisuke Nakane, Takayuki Nishizaka

**Affiliations:** 1 Department of Physics, Gakushuin University, 1-5-1 Mejiro, Toshima-ku, Tokyo, 171-8588 Japan; 2 Department of Physics, Tohoku University, Sendai, 980-8578 Japan

**Keywords:** Single-molecule biophysics, Motor protein function, Archaeal physiology

## Abstract

It is unknown how the archaellum—the rotary propeller used by Archaea for motility—works. To further understand the molecular mechanism by which the hexameric ATPase motor protein FlaI drives rotation of the membrane-embedded archaellar motor, we determined motor torque by imposition of various loads on *Halobacterium salinarum* archaella. Markers of different sizes were attached to single archaella, and their trajectories were quantified using three-dimensional tracking and high-speed recording. We show that rotation slows as the viscous drag of markers increases, but torque remains constant at 160 pN·nm independent of rotation speed. Notably, the estimated work done in a single rotation is twice the expected energy that would come from hydrolysis of six ATP molecules in the hexamer, indicating that more ATP molecules are required for one rotation of archaellum. To reconcile the apparent contradiction, we suggest a new and general model for the mechanism of ATP-driven rotary motors.

## Abstract (web summary)

Seiji Iwata et al. report protocols for visualizing and measuring the torque of the rotary propeller (archaellar) used by the Archea *Halobacterium salinarum*. They find that the estimated work done in a single rotation is more than expected and propose a new model for the mechanism of ATP-driven rotary motors.

## Introduction

Motile archaea swim using surface appendages called archaella (previously named “archaeal flagella”), which are thrust-generating rotating helical filaments analogous to bacterial flagella^1^. Although the bacterial flagellum consists of >30 different proteins, the archaellum is built from as few as 7 proteins, none of which are homologous to flagellar proteins^2^. Instead, the archaellum is evolutionarily related to bacterial type IV pili. Recent structural and functional studies of protein components have begun to provide us a model of archaellar architecture^1,3–5^. The mechanism of archaellar rotation, however, remains unclear, in part due to the lack of biophysical measurements that would constrain mechanistic models.

Components of the archaellar motor have been identified and biochemically characterized, providing the first steps to understand the mechanism of rotation^3^. Crucially, the archaellum is driven by ATP hydrolysis^6^ instead of proton flux as in the bacterial motor. The core archaellar motor is composed of the transmembrane protein FlaJ, ATPase FlaI, and associated FlaH. FlaI forms a hexamer^7,8^ that likely drives rotation, although it is unclear how FlaI generates torque. It is also unclear whether FlaI is static relative to the cell body or whether it rotates relative to the cell body together with the archaellum. FlaI has been shown to hydrolyze ATP^8^, and although the associated FlaH has an ATP binding motif^9^, there is no evidence that FlaH hydrolyzes ATP. Furthermore, no other archaellar components have been implicated as energy-transducing proteins, indicating that FlaI powers archaellar rotation alone, with a catalytic cycle involving sequential hydrolysis of six ATP molecules. Despite these biochemical insights, however, little is known of the physics of archaellar rotation.

We previously characterized archaellar function in the model organism *Halobacterium salinarum* using advanced fluorescent microscopy^10^. Challenges manipulating motor load, however, prevented us from measuring torque. To gain insights into the molecular mechanism underlying how FlaI drives rotation, we developed methods to measure archaellar motor torque. We found that archaellar torque remains constant at 160 pN·nm independent of rotation speeds between 0.5 and 30 Hz. Unexpectedly, the estimated work done in a single rotation was higher than the expected energy input that would come from the hexameric FlaI architecture, suggesting a model for the mechanism of archaellar motor rotation involving hydrolysis of more than one ATP molecule per FlaI subunit per full rotation.

## Results

### 3D rotation assay with beads

**Fig. 1:**
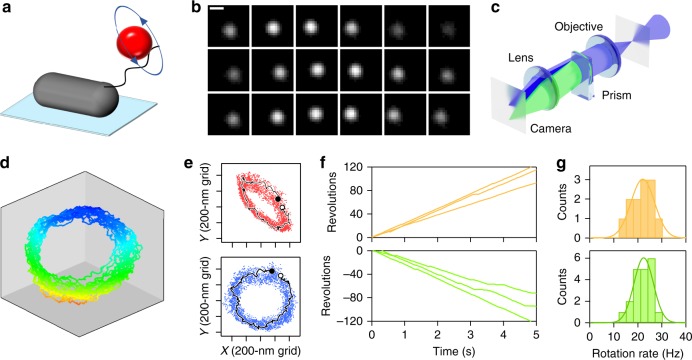
Rotation of a fluorescent polystyrene bead attached to an archaellum of a cell immobilized to a glass surface. **a** Schematic of the experimental set-up. **b** Sequential micrographs of a rotating 490-nm-diameter bead captured in 10 ms intervals. Scale bar, 0.5 μm. **c** Schematic illustration of our optical path set-up between the equivalent sample plane (eSP) of the optical microscope and CCD camera to track three-dimensional (3D) motion of a bead as point light source. Half of the blue light is diffracted by a wedge prism, which is located at the equivalent back focal plane of the objective, and so two separate images are focused on the camera plate. **d** 3D plot of location of the bead bound to an archaellum over time. Different colors represent height from the glass surface. **e** *xy*-trajectory of bead position (dots) and representative one rotation (thin black line; starting from the open circle and ending at the closed circle). Upper, raw data. Lower, same dataset reoriented in 3D so that the circular rotation is coincident with the *xy-*plane of the plot. **f** Examples of time course of rotation in clockwise (CW; upper) and counter-clockwise (CCW; lower) directions. **g** Histograms of rotational rate for CW (upper) and CCW (lower). In **d**–**g**, 210-nm-diameter beads were used as markers

Toward measuring archaellar motor torque, we developed protocols to attach markers to add viscous friction to rotating archaella. We previously described methods to immobilize cells on a glass surface to visualize rotation of archaella using fluorescent labeling^10^. Here we developed methods to attach fluorescent beads to *H. salinarum* archaella and visualized their rotation using a high-speed complementary metal oxide semiconductor (CMOS) camera (Fig. 1a). Beads rotated continuously (Fig. 1b and Supplementary Movie 1) both clockwise (CW) and counter-clockwise (CCW). As expected from previous results showing that archaella protrude obliquely from the cell^10^, trajectories of beads were ellipsoidal in the *xy*-plane (cf., Fig. 1e, upper panel). To check whether the bead orbit was the projection of a true circle and, if so, to estimate its radius, we applied three-dimensional (3D) tracking^11,12^ to the bead assay. A wedge prism at the equivalent back focal plane of the objective lens divided the image (Fig. 1c), enabling us to reconstruct the bead’s 3D trajectory from the change in relative displacement between the center positions of the two images, as previously described^11^ (Fig. 1d). We identified the plane on which the orbit became circular by a rotational search of the two angles in the polar coordinate (Fig. 1e; upper and lower panels are reconstructions of the same dataset viewed from different directions), enabling us to plot the rotation of each archaellum in its true plane of rotation (Fig. 1f). This measurement enabled quantification of archaellar rotation speed in both directions: 21.8 and 21.5 Hz for CW and CCW rotation, respectively, when using 210-nm-diameter beads as markers (Fig. 1g).

### Archaella rotation rate depends on load

**Fig. 2:**
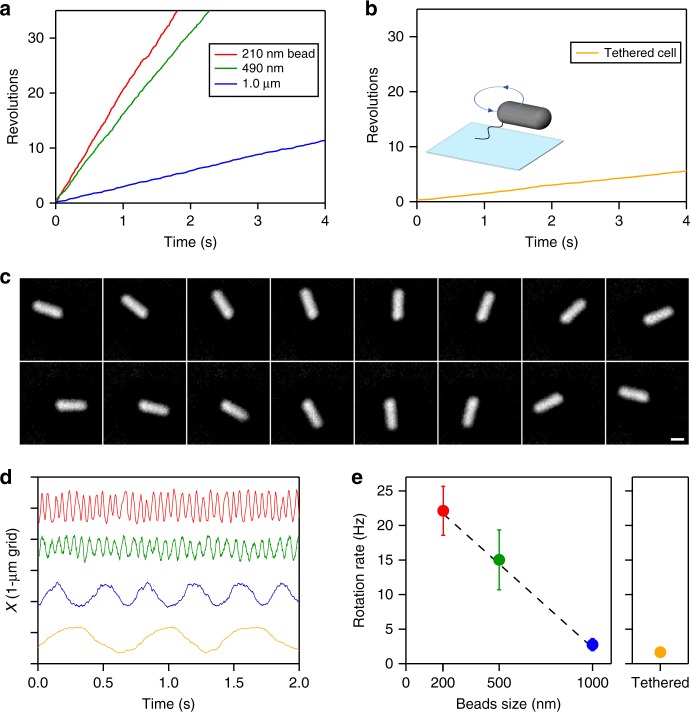
Archaella rotation rate is inversely proportional to load. **a** Example time courses of rotation of beads of different size. In all figures, red, green, blue, and yellow colors represent 210-nm, 490-nm, 1.0-μm diameter bead, and tethered cell, respectively. **b** An example time course of cell rotation in a tethered-cell assay. **c** Sequential phase-contrast micrographs of a tethered cell at 72 ms intervals. Image contrasts have been inverted for clarity. Scale bar, 1 μm. **d** Time courses of displacements of the center of markers. **e** Rotation rates of markers of different sizes. Error bars show standard deviations. The sources of data points are shown as values along the axis of ordinate in Fig. 3. Dotted line shows a linear fit, *V* = *A*/*ϕ* + *B*, where *V* is rotation rate; *ϕ* diameter of bead; *B* = 27 Hz, an intercept; and *A* = –0.024 Hz nm^−1^, a proportional coefficient

To determine archaellar rotation speeds under varying loads, we varied the sizes of the attached beads. Rotation rate decreased as we increased bead diameter (Fig. 2a) to a limit of 1 µm; larger beads did not rotate smoothly, perhaps due to intermittent collisions with the glass surface. To impose higher viscous loads, we therefore used the tethered-cell assay^10^ (see Fig. 2b). Two patterns of rotation were observed: smooth rotation without pauses as exemplified in Fig. 2a, b and Supplementary Fig. 1A; and saw-tooth rotation with unitary steps as previously observed^10^ and shown in Supplementary Fig. 1B. Reasoning that pauses relating to the slow rate-limiting chemical step(s) inhibit torque estimation (as with the difference between “rotation rate” and “stepping rate” in the F_1_-ATPase rotary motor^13,14^), we selected only cases with smooth rotation for further analysis. Rotation rate varied depending on marker diameter (Fig. 2d), decreasing as size increased (Fig. 2e), indicating that the archaellar motor in *H*. *salinarum* has an upper limit to its rotation rate of ∼25 Hz, as an extrapolation in Fig. 2e, which was reduced by the viscous drag of the attached beads. Additional loads further reduced rotation rates to ∼1 Hz while maintaining smooth unidirectional rotation. This observation is similar to the other, although unrelated, ATP-driven rotary motor, the F_1_-ATPase, which exhibits smooth rotation even when rotation rate slows to 1% the unloaded speed^14^.

### Estimation of torque

**Fig. 3:**
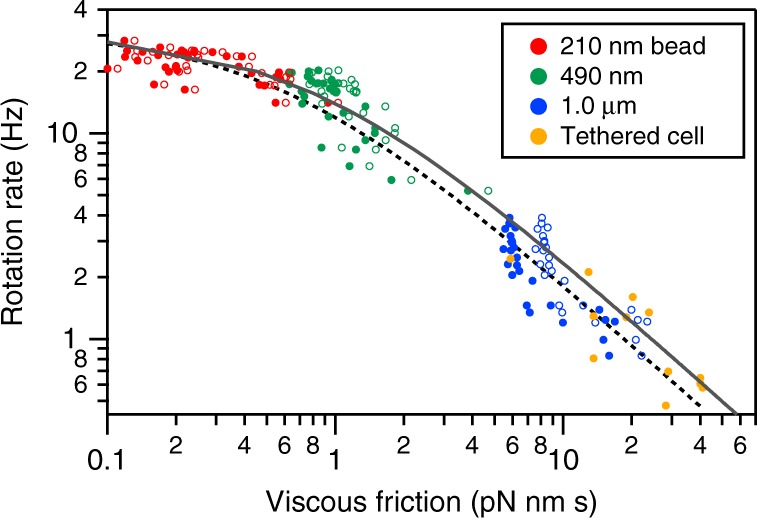
Relationship between rotation rate and viscous friction of different markers. Viscous friction, *γ*, was estimated from the size, shape, and radius of rotation of markers (either beads or tethered cell) and the measured viscosity of the solution, *η* = 1.35 × 10^−3^ Pa·s. Sample sizes are 32, 31, 26 and 12 for red, green, blue and yellow dots, respectively. Filled and open circles show raw data and the same dataset after correction, taking into account the effect of interaction with the surface. Two lines show fitting with the rate assuming that the motor produces a constant torque, *f*(*γ*) = *T*_a_/2*π*(*γ* + *γ*_a_), where *f* is the rotational rate; *γ* viscous friction; *T*_a_ the torque of the motor; and *γ*_a_ the constant. The dotted line is for raw data: *T*_a_ = 1.2 × 10^2^ pN·nm; *γ*_a_ = 6.0 × 10^−1^ pN·nm·s. The black line is for the dataset after correction: *T*_a_ = 1.6 × 10^2^ pN·nm; *γ*_a_ = 8.1 × 10^−1^ pN·nm·s. See the text for the interpretation of *γ*_a_ and its correction

It was unclear whether archaellar torque was constant under different loads. To calculate archaellar torque, therefore, we plotted rotation rate as a function of the viscous friction of attached markers. In conventional analyses of the bacterial flagellar motor, torque is plotted against speed in a linear scale^15–17^, based on the assumption that the viscous friction of single flagellum against the surrounding media can be calculated. Here, however, we assumed that we could not directly estimate torque in individual trajectories because archaellum lengths are unknown in each measurement, and eccentric archaellar filament rotation contributes non-negligible additional drag. We thus plotted rotation rate against the viscous friction of markers (Fig. 3). Rotation rate was directly determined from our measurements; to estimate viscous friction in each trajectory, we estimated the viscosity of the solution, *η*, to be 1.35 × 10^–3^ Pa**·**s using a viscometer and used this to calculate viscous friction from the shape and size of markers and their radius of rotation (see “Methods” section).

Using these results, we estimated archaellar motor torque. If the motor produces constant torque *T*_a_ in any condition regardless of viscous drag, the rotation rate, *f*, will be inversely proportional to the viscous friction, *γ*, as *f* = *T*_a_/2*πγ*. Our data confirmed this relationship under high loads, but indicated an additional friction factor, *γ*_a_, that contributed apparent additional drag that becomes dominant at fast rotation under low loads. This parameter therefore represents a frictional drag factor in addition to that of the attached bead. Incorporating *γ*_a_, we found that *f* = *T*_a_/2*π*(*γ* + *γ*_a_), with *T*_a_ = 120 pN·nm and *γ*_a_ = 0.60 pN·nm·s for our dataset (indicated by the dotted line in Fig. 3).

We refined *T*_a_ and *γ*_a_ values by correcting for the interaction of the bead with the glass surface, as the surface suppresses flow induced by the moving bead, leading to increased frictional drag. We corrected for this effect using correction factors estimated based on hydrodynamic calculation^18^, resulting in a shift of data points to the right (see black line in Fig. 3; see “Methods” section for the factors). This correction enabled us to refine our motor torque calculation to be 160 pN·nm. Similarly, we re-estimated *γ*_a_, the additional viscous friction parameter, to be *γ*_a_ = 0.81 pN·nm·s. The formulation based on both *T*_a_ and *γ*_a_ is applicable as shown in the apparent torque plot against rotation rate (Supplementary Fig. 2A, B). Constant torque was shown after subtraction of the contribution of *γ*_a_ (Supplementary Fig. 2C) in the range of our measurements, which contrasted with the torque/speed behavior of the bacterial flagellar motor in which torque declines at lower loads (see Fig. 5 in ref. ^15^ and Fig. 4 in ref. ^17^).

**Fig. 4:**
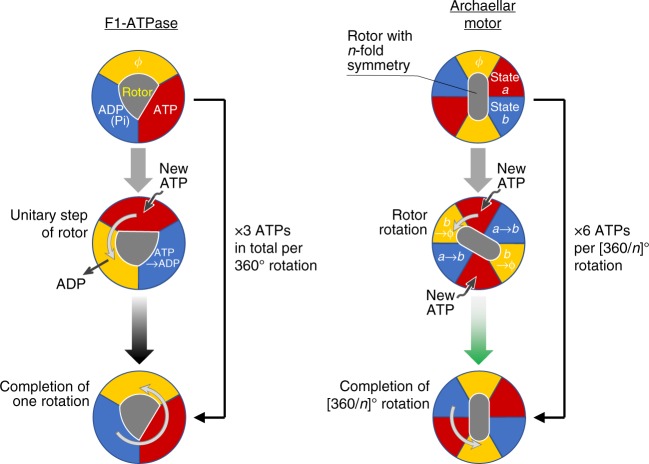
A model for the mechanism of ATP hydrolysis by the archaellar motor based on similarity to the F_1_-ATPase. Left, The mechanism of the F_1_-ATPase that we have previously demonstrated^24–26, 33^. Two of the three catalytic subunits are occupied by nucleotides, either ATP (red) or ADP (blue), while the remaining subunit is empty (yellow). In this schematic, the positions of three subunits are fixed while the rotor rotates against them. Because the three catalytic subunits are cooperative, each subunit undergoes a determinate cycle through “empty state (*ϕ*)” → “ATP binding state” → “post-hydrolysis state” with each discrete 120° rotation. As a consequence, one F_1_-ATPase with three catalytic subunits consumes three ATPs per rotation, with a single ATP hydrolysis per subunit per 360° rotation. Right, our model for the mechanism of ATP hydrolysis-driven archaellar rotation. The principles are similar to the F_1_-ATPase, except the catalytic cycle for each catalytic subunit repeats every [360/*n*]° rotation (green arrow) due to the *n*-fold symmetry of the rotor and six active sites. Note that the figure is conceptual: our model does not depend on a specific structure and is not intended to be structurally explicit. For simplicity, the schematic is illustrated with the assumption of *n* = 2, which is consistent with our results and observations of two-fold symmetry in the structure of the cyclic FlaI hexamer^8^

## Discussion

We have described method development to measure the torque of the *H*. *salinarum* archaellar motor. We found that the motor’s torque output was 160 pN·nm regardless of rotation rate, in contrast to the bacterial flagellar motor in which torque is reduced for lower loads. Strikingly, as discussed below, this torque is approximately twice the anticipated torque that hydrolysis of six ATP molecules per full rotation could generate. We also identified an additional viscous friction factor, *γ*_a_.

What is the physical basis for the additional *γ*_a_ factor? We speculated two models: in Model A, *γ*_a_ represents the frictional drag coefficient of the archaellum itself, as seen with the viscous drag measured for bacterial flagella;^15–17^ in Model B, *γ*_a_ is due to the mechanochemical chemical cycle, independent of load. To assess Model A, we calculated the drag coefficient of an archaellum, approximating it as a helical screw of appropriate dimensions^10^. We calculated an estimated archaellar drag coefficient of 0.7 pN·nm·s (see “Methods” section), consistent with our *γ*_a_ value of 0.81 pN·nm·s. We also confirmed the length of archaella on cells prepared for our flow chamber using electron microscopy (Supplementary Fig. 3). Archaellar filaments averaged 2.6-μm long immediately after shearing, re-growing to 4.6 μm after 2 h. The parameter used in the above is within this range. Model B, in which rotation rate with a constant torque has a rate-limiting step, *f*(*γ*) = (1/*f*_noload_ + 2*πγ*/*T*_a_)^−1^ (see Fig. 2 in ref. ^14^), is also consistent with our observations. Key chemical step(s), such as hydrolysis of ATP or ATP binding, are the rate-limiting steps, resulting in *f*_noload_ as an upper limit for rotation. Also, the above equation turns to the same formulation in the legend of Fig. 3 with the assignment of *γ*_a_ = *T*_a_/2*πf*_noload_, and so discrimination between Models A and B is not possible in the fitting curve in Fig. 3. To assess Model B, it may be necessary to construct a ghost model of archaea analogous to that of the ATP-driven gliding machinery in *Mycoplasma mobile* (refs. ^19,20^; the ghost model enabled us to change the concentration of ATP), although we anticipate that this may be technically challenging.

Our results show that the archaellar motor produces constant torque independent of load, an observation that hints at the underlying molecular mechanism. Constant torque is also seen in the F_1_-ATPase (ref. ^13^), the soluble subcomplex of F_o_F_1_ ATP synthase but not the bacterial flagellar motor or linear motors driven by ATP hydrolysis, such as myosin, in which the force per motor is concave in relationship to load^21^, or kinesin, which linearly changes speed with load^22,23^. In the case of the bacterial flagella motor, torque is constant up to a certain speed, after which it drops to zero, behavior believed to be a hallmark of the torque generation mechanism^15,17^. Although FlaI and the α/β-subunits of the F_1_-ATPase (which include the catalytic site) are not closely related, their common architecture containing multiple symmetrically arranged catalytic sites in a cylinder evidently results in a common rotary mechanism.

Unexpectedly, however, our result indicates that motor torque exceeds that anticipated by hydrolysis of six ATP molecules per rotation—i.e., resulting from one ATP hydrolyzed per FlaI subunit. Our estimated torque of 160 pN·nm (Fig. 3) requires work for a single rotation to be 2*πT*_a_ ~ 1000 pN·nm. This estimated value exceeds the work from hydrolysis of six ATP molecules (one per FlaI subunit), 6 × Δ*G*_ATP_ ~ 5 × 10^2^ pN·nm, given that Δ*G*_ATP_ is 8 × 10 pN·nm in cells^13^. If each FlaI subunit hydrolyzed a single ATP molecule per full archaellar rotation, it follows that the archaellar motor would have a paradoxical apparent efficiency of nearly 200%.

The simplest explanation for this apparent paradox is that FlaI uses more than six ATP molecules to power a single archaellar rotation. The analogous F_1_-ATPase^24–26^ has three catalytic subunits cores, and three ATPs are hydrolyzed per turn of the rotor, which approaches ∼100% efficiency^13^. Key to the mechanism is the strict cooperativity of three catalytic subunits, with the chemical state in one subunit returning to its original state after a full rotation of the rotor. This coincides with the asymmetric structure of the F_1_-ATPase shaft^27^, as a particular side always faces just one of the three subunits in the specific chemical state (Fig. 4, left), confirmed through single-molecule observations using fluorescently labeled ATP (see Fig. 2e in ref. ^24^). Three ATP molecules are thus hydrolyzed per 360° rotation. In the archaellar motor, we hypothesize that the cooperativity between subunits is similarly strict, but unlike the asymmetric F_1_-ATPase shaft, we propose that the structure against which FlaI rotates has *n*-fold symmetry (whether FlaI is part of the archaellar stator or rotor) (Fig. 4, right). Parts of the *n*-fold symmetric rotor will therefore always face *n* FlaI subunits in the corresponding specific state, resulting in *n* active subunits at any time. Therefore, a 360° rotor rotation would induce hydrolysis of 6 × *n* ATPs by FlaI. To fulfill the archaellar work requirement of 1000 pN·nm, *n* must be >1. An *n* value of 2 is a good match for these requirements, with 12 ATP molecules hydrolyzed per full rotation, providing work of 1 × 10^3^ pN·nm, and corresponding to an efficiency of 100%. Larger submultiples of six are also possible values of *n* (*n* = 3 and 6 would give efficiency of 67 and 33%, respectively), but a precedent for a value of 2 for *n* is set by structural studies: the archaellar component FlaJ (ref. ^1^) may form a dimer, and the FlaI hexamer has two-fold symmetry instead of a pure six-fold symmetry^8^, as does its homolog PilB from the related type IV pilus^28^, suggesting further significance of an as yet to be discovered two-fold element. Our torque measurement and energy estimation contribute critical information toward establishing how the archaellar motor works.

## Methods

### Strains and cultivation

*H*. *salinarum* NRC1 (ATCC 700922) (ref. ^29^) was prepared as previously described^30^ with modifications^10^: Cells were grown at 45 °C on agar plates (3.4 M NaCl, 0.12 M MgCl_2_, 0.12 M MgSO_4_, 0.08 M KCl, 0.5% (w/v) casamino acid, 0.002% (w/v) biotin, 0.005% (w/v) thiamine hydrochloride, 0.01% (w/v) L-tryptophan, 0.01% (w/v) uracil, 10 mM HEPES-NaOH (pH 7.0) and 1% (w/v) agar (Wako 010–08725)); and red-colored colonies were picked with the tip of a micropipette and suspended in motility buffer (1.2 M NaCl, 0.7 M MgCl_2_, 10 mM HEPES-NaOH (pH 7.0)).

### Bead assay

All experiments were performed at room temperature, required for stability of the observation system for nanometer-resolution during recording. Streptavidin (Sigma-Aldrich) was conjugated with either carboxylated polystyrene beads (Polysciences) with sizes between 490 nm and 1.0 μm in diameter by a standard procedure using 1-ethyl-3-(3-dimethylaminopropyl)carbodiimide^31^ or amino-modified 210 nm beads (Polysciences) conjugated through biotin-(AC_5_)_2_-Sulfo-OSu, as previously described^25^. The flow chamber was comprised of two coverslips (No. 1, 0.12–0.17-mm thickness; Matsunami Glass): 18 × 18 mm^2^; and 24 × 36 mm^2^ glow-discharged with a hydrophilic treatment device (PIB-10; Vacuum Device). Two pieces of double-sided tape cut to approximately 5 mm wide and 30 mm in length serves as spacers for the two coverslips. The resulting flow chamber had an internal volume of ~7 μl (ref. ^10^). Biotinylated cells^10^ were mechanically sheared by passing 30 times in and out of a pipette tip and directly infused into the flow chamber in motility buffer. After incubation for 5 min, the chamber was rinsed with 20 μl motility buffer to remove unbound cells. Beads were subsequently infused and unbound beads washed off after incubation for 10 min. We did not estimate torque from data in which rotation exhibited periodic changes in speed (typically every 60°, Supplementary Fig. 1B).

### Microscopy

An inverted microscope (Ti-E; Nikon Instruments) was equipped with a ×100 objective (Plan Fluor, N.A. 1.3; Nikon Instruments), a CMOS camera (DMK 23UX174; Imaging Source) set to the camera port in an eye-piece unit, a highly stable customized stage (Chukousha), and an optical table (RS-2000; Newport). The left camera port of the microscope was connected to a high-speed camera (HDR-20000; Digimo) through a custom-made 3D tracking optical component, in which the wedge prism can be adjusted along the three axes in a blocked box. Images were recorded typically at 0.5-ms intervals with magnification to provide 98 × 98 nm^2^ pixel size. Data were analyzed using Igor Pro (Wave Metrics) and ImageJ.

### Calculation of drag coefficients of markers and archaella

Solution viscosity was determined using a viscometer equipped with sensors driven by electromagnets to vibrate with a constant sine-wave vibration in reverse phase (SV-10; A&D Company). Viscosity measurements of buffer were taken ranging between 21.3 and 53.6 °C with calibration using distilled water. Viscosity was inversely proportional to temperature, and *η* of the solution was estimated to be 1.35 × 10^–3^ Pa**·**s under our measurement conditions. For beads, the equation *γ* = 8*πηr*^3^ + 6*πηrR*^2^ was applied where *r* is radius of bead and *R* the rotation radius^14,32^. For corrections to estimate the precise torque in Fig. 3, we assumed the bead moved parallel to the glass surface and that the gap between the glass surface and the bead was equivalent to the width of the cell, 0.5 μm. Correction factors were thus calculated to be 1.4, 1.2, and 1.1 for 1.0-, 0.49-, and 0.21-μm beads, respectively (chapter 7–4 in ref. ^18^). For the tethered-cell assay, the drag coefficient of a rod, *γ* = 1/3 × *πηL*^3^ × [ln(*L*/2*r*) − 0.66]^−1^, was applied where *r* is the radius of the rod cross-section and *L* is the length of the rod. In our measurements, the archaellar pivot point is located at the end of the cell, and thus $\frac{1}{2}\gamma$ with *L* = 2 × (cell length) was used. To discuss the contribution of viscous drag of a single archaellum, the equation, *γ* = 2*πηb*^2^*L*(2*p*^2^ + 4*π*^2^*b*^2^) × (*p*^2^ + 4*π*^2^*b*^2^)^−1^ × [ln(2*p*/*r*) − 0.5]^−1^ (ref. ^17^), was applied to estimate its frictional drag coefficient. *γ* was calculated to be 0.7 pN·nm·s with structural parameters^10^ *b* = 0.22 μm (archaellum helix radius), *L* = 4.3 μm (archaellum length), *p* = 2.1 μm (archaellum helix pitch), and *r* = 7 nm (archaellum radius).

### Reporting summary

Further information on research design is available in the Nature Research Reporting Summary linked to this article.

## Supplementary information

Description of Additional Supplementary Files

Supplementary Information

Reporting Summary

Supplementary Movie 1

## Data Availability

The data that support the findings of this study are available from the corresponding author upon reasonable request.
