# Supplementary material for: Motor torque measurement of Halobacterium salinarum archaellar suggests a general model for ATP-driven rotary motors
Source: Commun Biol. 2019 May 24;2:199. doi: 10.1038/s42003-019-0422-6 (PMC6534597; doi:10.1038/s42003-019-0422-6)
Supplement: Supplementary file 1 — Description of Additional Supplementary Files [file 42003_2019_422_MOESM1_ESM.pdf]

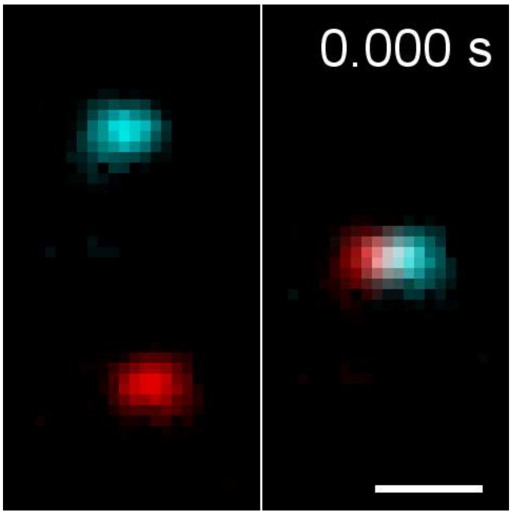

Supplementary Movie 1. Rotation of a fluorescent bead bound to an archaellum. A bead image was split in two (cyan and red in the *Left panel*) as shown in Fig. 1C, and integrated into one image (*Right*). The height of the bead from the glass surface is greatest when the two images superimpose as shown in *Right panel* (appearing white); the images of lower beads do not align, and the height can be calculated from the displacement of the twin images. The 3-D trajectory of bead rotation can be reconstructed from the relative displacement between the center positions of the two images. Scale bar, 1 μm.
