# Supplementary material for: Motor torque measurement of Halobacterium salinarum archaellar suggests a general model for ATP-driven rotary motors
Source: Commun Biol. 2019 May 24;2:199. doi: 10.1038/s42003-019-0422-6 (PMC6534597; doi:10.1038/s42003-019-0422-6)
Supplement: Supplementary file 2 — Supplementary Information [file 42003_2019_422_MOESM2_ESM.pdf]

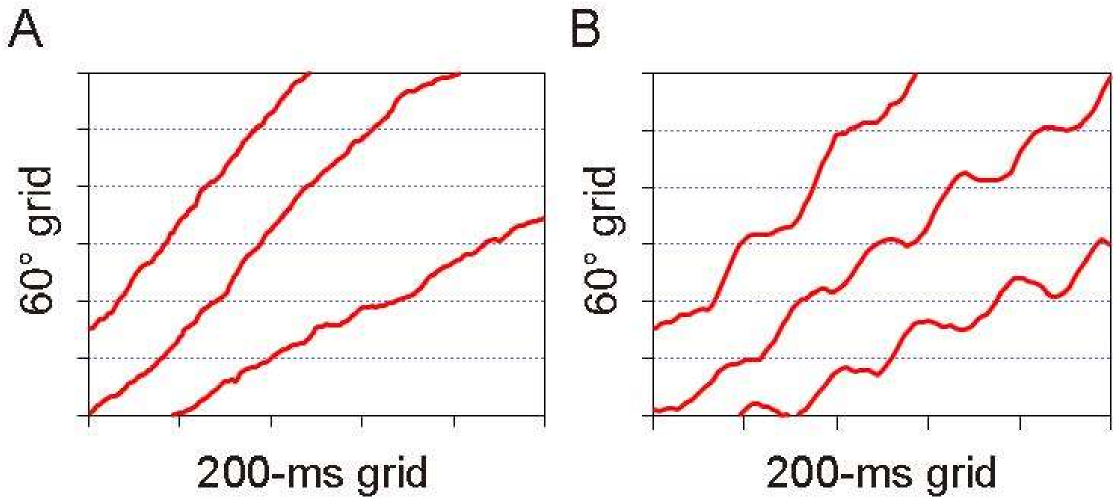

Figure S1 | Iwata *et al*.

Supplementary Figure 1. Examples of two patterns of rotation. (*A*) Smooth rotation without pauses in a tethered-cell assay. (*B*) Saw-tooth rotation with unitary ~60° steps as previously observed (Kinosita *et al.*, *Nature Microbiology*, 2016). The frequency of observation of the two patterns was similar.

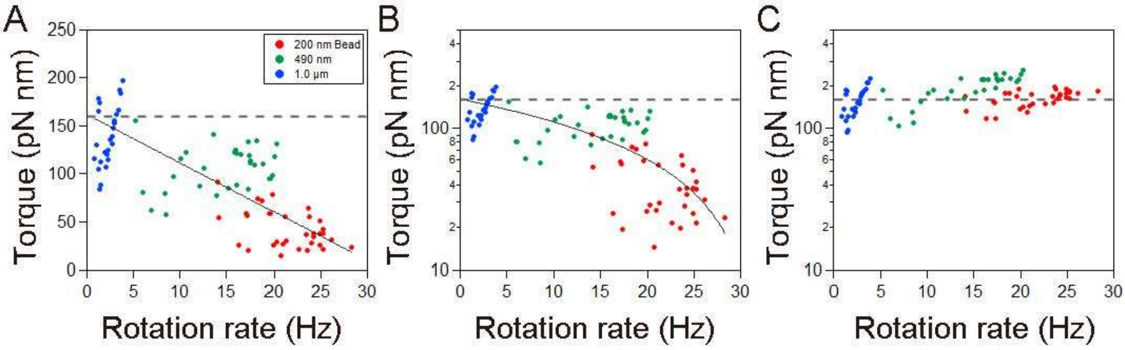

Figure S2 | Iwata *et al*.

Supplementary Figure 2. Relationship between torque and rotation rate on the different markers. The dataset was taken from open circles in Fig. 3, which contain the correction taking into account the effect of the interaction of the bead with the surface. (*A*) Linear plot; (*B*) Semi-log plot; and (*C*) Semi-log plot after the subtraction of the effect of γ_a. Dashed lines represent T_a = 1.6×10^2 pN·nm, and black lines in *A* and *B* show the fitting with a linear relationship with γ_a = 8.1×10^{-1} pN·nm·s.

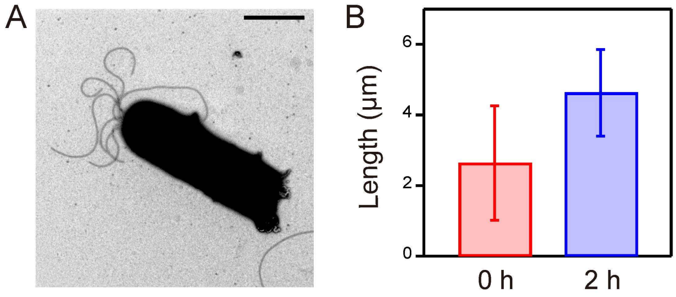

Figure S3 | Iwata *et al*.

Supplementary Figure 3. Lengths of archaella after shearing. (*A*) Image of a cell taken by electron microscopy negatively stained using ammonium molybdate. Scale bar, 1 µm. (*B*) Histograms of archaella length measured from electron micrographs. Bars represent standard deviations. *Left*, length just after shearing, defined as time 0 (n = 20). *Right*, Length two hours after shearing (n = 15).
